# Dual HDAC and PI3K Inhibitor CUDC-907 Inhibits Growth of Pleural Mesothelioma: The Impact of Cisplatin Sensitivity and Myc Expression

**DOI:** 10.3390/cells14201599

**Published:** 2025-10-15

**Authors:** Luca Hegedüs, Silvia Qaisieh, Christian Stülpnagel, Yavar Ganjeh Khor Dezfouli, Winny Tambo, Fabian Doerr, Natalie Baldes, Dirk Theegarten, Martin Schuler, Servet Bölükbas, Balazs Hegedüs

**Affiliations:** 1 Department of Thoracic Surgery, University Medicine Essen—Ruhrlandklinik, West German Cancer Center, University Duisburg-Essen, Tüschener Weg 40, 45239 Essen, Germany; luca.hegedues@rlk.uk-essen.de (L.H.);; 2 Institute of Pathology, University Medicine Essen, West German Cancer Center, University Duisburg-Essen, Hufelandstraße 55, 45147 Essen, Germany; 3 Department of Medical Oncology, University Medicine Essen, West German Cancer Center, University Duisburg-Essen, Hufelandstraße 55, 45147 Essen, Germany; 4 National Center for Tumor Diseases (NCT), NCT West, 45147 Essen, Germany

**Keywords:** pleural mesothelioma, HDAC inhibitor, PI3K inhibitor, dual inhibitor, fimepinostat, c-Myc, cisplatin

## Abstract

**Objectives:** Pleural mesothelioma (PM) is a rare cancer that often develops after a decades-long latency period and confers a grim prognosis. Novel, biomarker-based therapeutic modalities are expected to improve the outcome of patients with advanced PM. CUDC-907 (fimepinostat) is a dual inhibitor that affects both histone deacetylases and PI3K enzymes. Its antitumor activity was described in several cancer types, but it has not yet been explored in PM. **Materials and Methods:** The sensitivity of 22 PM cell lines—including 18 models established in our laboratory—to cisplatin and CUDC-907 was determined using a cell viability assay. BAP1, PTEN, and c-Myc expression, as well as *MYC* copy number variation, were measured. The effect of combination treatment with cisplatin was assessed with cell viability, cell cycle, and 3D spheroid formation assays. **Results:** Most PM cell lines were sensitive to CUDC-907 treatment, and the CUDC-907 response was significantly higher in cell lines with higher c-Myc expression due to *MYC* copy number gain or amplification. Importantly, all cisplatin-insensitive cell lines were sensitive to CUDC-907. Combination treatment with cisplatin synergistically decreased cell viability and induced G2/M arrest or cell death. We tested cisplatin-sensitive P31WT and cisplatin resistant P31cis isogeneic pair and found that in both 2D and 3D assays the cisplatin-resistant cells showed a higher sensitivity to CUDC-907 single treatment. Combining CUDC-907 with cisplatin further decreased cell growth even in cisplatin-resistant cells. **Conclusions:** The majority of PM cell models are sensitive to CUDC-907, which may be a potent therapeutic agent in PM.

## 1. Introduction

Pleural mesothelioma (PM) is a rare type of cancer arising mainly after exposure to asbestos. It develops after a decades-long latency period and is associated with a very grim prognosis [1]. The therapeutic options depend on disease stage and histological subtypes. For late-stage disease, chemo- and/or immune checkpoint inhibitor therapy is approved. For early-stage disease, the multimodal therapy can include surgery, radiotherapy, and chemo- or immune checkpoint inhibitor therapy. However, median survival is between 9 and 18 months, partially due to intrinsic or acquired therapeutic resistance [2]. In chemotherapy, PM patients receive cisplatin or carboplatin in combination with pemetrexed. Mutation or deletion of BRCA1-associated protein 1 (BAP1) has been associated with therapy resistance, just as increased metallothioneins and decreased expression of CAAT/enhancer binding protein (C/EBP)-β LIP [3,4,5]. Both intra- and intertumoral heterogeneity are very high in mesotheliomas due to their polyclonal origin and very long development [6]. For this reason, novel, biomarker-based therapeutic modalities are required to improve patient outcomes.

CUDC-907 (fimepinostat) is a small molecule with an acceptable safety profile that has dual effects, as it inhibits both histone deacetylases (class I and II) and PI3K enzymes (class I α, β, δ) [7]. CUDC-907 has not yet received full approval by the FDA; however, the agency granted a fast track designation to this drug in 2018 for adult diffuse large B-cell lymphoma (DLBCL) patients who have already received two or more lines of systemic therapy. CUDC-907 is composed of the hydroxamate moiety of the HDAC inhibitors and a PI3K inhibitor skeleton and induced higher growth inhibition and a stronger proapoptotic effect as a single agent than HDAC and PI3K inhibitors [8]. Single-agent treatment with both PI3K and HDAC inhibitors is accompanied by toxicity and induced drug resistance. Dual inhibitors aim to overcome these challenges by targeting multiple pathways as treatment with multiple single-agent drugs, but with less toxicity and with limited drug–drug interactions [9]. The antitumor activity of CUDC-907 was demonstrated in lymphomas as well as in solid tumors [10]. In prostate cancer cells, CUDC-907 treatment induced DNA damage and apoptosis, while in the PDX model, it decreased tumor growth [11]. Similarly, in small cell lung cancer (SCLC) cell lines, CUDC-907 treatment evoked cell cycle arrest and decreased DNA double-strand break (DSB) repair. Combination treatment with the PARP inhibitor olaparib had a synergistic effect both in vitro and in PDX mouse models [12].

In this study and in several others, it was found that c-Myc and its paralogs are downregulated by CUDC-907 and which was coupled with reduced cell growth and proliferation. This was also demonstrated in models where, due to *MYC* translocation or amplification, c-Myc expression is strongly increased. It was found that inhibition of HDAC activity and PI3K pathway activation alone decreases c-Myc protein expression, while CUDC-907 treatment reduces *MYC* gene transcription, enhances c-Myc protein proteasomal degradation, and can alter the transcriptional regulation of certain Myc-dependent genes [13]. In pleural mesothelioma, *MYC* expression is often upregulated. In an early study of PM tissue samples and non-malignant pleural mesothelium, c-Myc staining was present both in the nucleus and the cytoplasm, but in a higher proportion in the malignant samples [14]. Later analysis of a large PM cell panel showed that 78% of the cells had an increased expression of *MYC1* or *MYC2,* and Myc inhibitor treatment decreased cell proliferation [15]. *MYC* and the *PVT1* genes are located in the 8q24 chromosomal region, and they are often coamplified. *MYC* copy number gain (CNG) was found to be frequent both in PM cell lines and tissue samples; however, *MYC* expression did not correlate with clinicopathological features. Downregulation of *MYC* or the long non-coding RNA *PVT1* decreased proliferation and enhanced the cisplatin sensitivity of the PM cell line MSTO-211H [16]. Moreover, CUDC-907 treatment was effective in platinum-resistant MYC-expressing SCLC cell lines. It was demonstrated that *MYC* expression was strongly increased by platinum treatment both in vitro and in vivo, and in combination with standard of care chemotherapy, CUDC-907 treatment decreased tumor growth and synergistically increased survival in the SCLC mouse model [17].

CUDC-907 also reduced cancer cell migration, invasion, and spheroid growth in esophageal cancer, bladder cancer, hepatocarcinomas, and pediatric neuroblastoma [18,19,20,21]. It was found that CUDC-907 can enhance the effect of several therapeutic agents. In acute myeloid leukemia, combination treatment of CUDC-907 with Wee1 inhibitor adavosertib showed a synergistic effect in both AML cell lines and primary specimens by enhancing the adavosertib-initiated DNA damage [22]. In diffuse intrinsic pontine glioma, CUDC-907 and gemcitabine showed synergistic antitumor efficacy both in cell lines and xenograft models, as CUDC-907 impeded the gemcitabine-induced activation of NF-κB signaling [23]. Phase 1 and 2 trials among patients with DLBCL indicated that Myc alteration is a predictive biomarker for therapy response in this disease, and combination treatment with monoclonal antibody rituximab enhanced the antitumor effect [24,25].

The effect of CUDC-907 on PM cell lines has not yet been explored. In preclinical studies, HDAC inhibitors showed substantial efficacy both in PM cell lines and mouse models [26]. However, in a phase 3 randomized clinical trial of PM patients who already showed progression after first- or second-line systemic therapy, vorinostat as a single agent did not prolong survival [27]. More recently, it was found that HDAC inhibition can potentiate the effect of standard-of-care treatments in PM. HDAC inhibitor entinostat increased the sensitivity of PM cell lines to both cisplatin and radiation, and combination treatment with cisplatin and entinostat had a synergistic growth inhibitory effect through higher platinum uptake [28]. Additionally, combined treatment with hypomethylating agent decitabine and HDAC inhibitor valproic acid increased expression of immune checkpoint inhibitors on PM cells, while valproate also increased the cytotoxicity of blood-derived monocytes towards PM cells in cocultures [29,30]. Increased activation of the PI3K/AKT/mTOR pathway was described in PM cells in comparison to non-malignant mesothelial cells due to the activation of receptor tyrosine kinases [31]. In a recent study, it was found that expression of PI3K-δ was moderate or strong in 74% of PM tissue samples, and this was coupled with significantly shorter survival. PI3K-δ-specific inhibitor roginolisib initiated apoptosis in PM cells and enhanced the effect of AKT and mTOR inhibitors. Furthermore, in combination with chemotherapy and immunotherapy, it increased the effector function of antitumor immune cells [32]. Additionally, in a phase 1 trial among advanced mesothelioma patients, the class I PI3K isoforms and mTORC1/2 inhibitor LY3023414 were evaluated as a single agent, and it showed an acceptable safety profile but limited activity [33].

Based on these findings, we hypothesized that the combined targeting of HDACs and the PI3K/AKT/mTOR pathway can inhibit the growth of pleural mesothelioma. We used a panel of 22 PM cell lines and analyzed their sensitivity to CUDC-907 using 2D and 3D models and explored the effect of the combination treatment with the standard-of-care platinum-based therapy.

## 2. Materials and Methods

### 2.1. Cell Lines and Compounds

We used 22 pleural mesothelioma cell lines in our experiments. A total of 18 PM cell lines were established in our laboratory from the pleural effusion of PM patients as described earlier [34]. This study was approved by the Ethics Committee at the University Hospital Essen (#18–8208-BO), and patients provided informed consent. Patient characteristics, BAP1, and PTEN expression of the cell line cohort are summarized in Supplementary Table S1. This cell line panel is representative of the general mesothelioma cohorts in terms of gender, age, asbestos exposure, and histotype distribution, as well as reflects the molecular heterogeneity regarding BAP1 and PTEN loss. The SPC111 cell line was acquired from Sigma-Aldrich (St. Louis, MO, USA). The SPC212 cell line was kindly provided by Prof. R. Stahel (University of Zurich, Zurich, Switzerland) [35]. P31 wild type and its cisplatin-resistant pair were a kind gift from Prof. K. Grankvist (University of Umea, Umea, Sweden) [36,37]. The SPC111 and SPC212 originated from human PM tumors with biphasic, the P31 cell lines with epithelioid morphology. All cell lines were authenticated by Single Nucleotide Analysis by Multiplex Cell Line Authentication (Multiplexion, Heidelberg, Germany). Representative phase contrast images of all 22 lines are shown in Supplementary Figures S1 and S2. The cells were kept in DMEM media containing 10% FBS and 1% penicillin-streptomycin in a humidified atmosphere with 5% CO_2_ and at 37 °C. CUDC-907 dissolved in DMSO at a 10 mM concentration was purchased from MedChemExpress (Monmouth Junction, NJ, USA), and cisplatin (Accord, Munich, Germany) was dissolved in 0.7% NaCl.

### 2.2. Cell Viability Assay

The Sulforhodamine B (SRB) assay was used to determine the effect of CUDC-907 on the cell viability of PM cells. First, 3000 to 8000 cells were seeded on the inner wells of 96-well plates, then the cells were incubated for 24 h at 37 °C. Afterwards, the cells were treated with increasing concentrations of CUDC-907 (1 nM, 10 nM, 30 nM, 0.1 µM, 0.3 μM) for 72 h. Upon combination treatment with cisplatin, we treated the cells with CUDC-907 (1 nM, 10 nM, 30 nM, 50 nM) and cisplatin (2 μM, 4 μM, 6 μM, 8 μM) in all combinations. Then the plate was washed once with PBS, and the cells were fixed by precipitating total protein by adding to each well ice-cold, 6% trichloroacetic acid (TCA). After we incubated the plate for one hour at 4 °C, we washed the TCA away by rinsing the plate with distilled water five times and dried the plate for 1–2 days. In order to analyze the plate, first, we stained it with 0.4% SRB (Sigma-Aldrich, St. Louis, MO, USA) for 15 min. Then we washed the plate several times with 1% acetic acid to remove excess dye and dried the plate for 1–2 h. Afterwards, we dissolved the protein-bound dye with 10 mM Tris buffer and measured optical density with a microplate reader (Infinite F50 Plate Reader, Tecan, Männedorf, Switzerland) at 570 nm. We used CompuSyn software version 1.0 (ComboSyn Inc., Paramus, NJ, USA) to calculate half-maximal inhibitory concentration (IC50) and combination index (CI) [38]. To calculate CI values, we used the average of three independent viability measurements. CI values indicate drug combinational interactions, synergism (CI < 0.9), additive effect (CI is between 0.9 and 1.1), or antagonism (CI > 1.1).

### 2.3. Immunoblot Analysis

We seeded PM cells on 6-well plates (1.5–3 × 10^5^ cells/well) and let the cells attach overnight. Then CUDC-907 (20 nM, 30 nM, 50 nM, 60 nM) and Cisplatin (4 μM) treatments were applied either as a single treatment or in combinations. Protein isolation was performed after 24 or 72 h long treatment. First, we washed the plate twice with PBS, then we added 1 mL ice-cold 6% TCA solution to each well to precipitate cellular protein. This was followed by an incubation at 4 °C for 1–6 h, then the precipitated protein was scraped and collected. Samples were centrifuged for 10 min at 8000 rpm at 4 °C. Subsequently, the pellets were dissolved in electrophoresis sample buffer (62.5 mM Tris-HCl, pH 6.8, 2% SDS, 10% glycerol, 5 mM EDTA, 125 mg/mL urea, 100 mM dithiothreitol). Equal amounts of total protein (20 or 30 μg) from each sample were loaded on 7.5%, 10% or 15% acrylamide gels. Rabbit monoclonal anti-Acetyl-Histone H3 (Lys9) (C5B11) (Cell Signaling, 9649, 1:1000), rabbit polyclonal anti-PARP (Cell Signaling, 9542, 1:1000), rabbit monoclonal anti-c-Myc antibody (Cell Signaling, 5605, 1:1000), rabbit monoclonal anti-phospho-Akt (Ser473) (193H12) antibody (Cell Signaling, 4058, 1:1000), rabbit monoclonal anti-phospho-ERK (Ser473) (193H12) antibody (Cell Signaling, 4058, 1:1000), rabbit monoclonal anti-E-cadhrein (24E10) (Cell Signaling, 3195, 1:1000), rabbit monoclonal anti-Vimentin (D21H3) (Cell Signaling, 5741, 1:1000), rabbit monoclonal anti-beta-Actin (Cell Signaling, 4970, 1:1000) and rabbit polyclonal anti-beta-tubulin (Abcam, Cambridge, United Kingdom, ab6046, 1:2000) primary antibodies were used for immunostaining. For detection, HRP-conjugated anti-rabbit and anti-mouse secondary antibodies (Jackson ImmunoResearch, West Grove, PA, USA, dilution 1:10,000) were used and luminography was performed with Pierce ECL Western blotting Substrate (Thermo Scientific, Waltham, MS, USA).

### 2.4. Cell Number and Cell Cycle Analysis

We seeded the PM cells on 6-well plates (1.5–3 × 10^5^ cells/well), and the next day, CUDC-907 (20 nM, 60 nM) and Cisplatin (3 μM, 4 μM) treatments were applied either as a single treatment or in combinations. Cell number and cell cycle analysis were performed after 72 h. First, cells from the supernatant were collected, and then attached cells were trypsinized. Total cell number was determined with the Viability and Cell Count assay of the NucleoCounter NC-3000™ system (Chemometec, Allerod, Denmark). Afterwards, each sample was washed once with PBS, and lysis buffer treatment (Solution 10, 910-3010, Chemometec) supplemented with the DAPI staining solution (Solution 12, 910-3012, Chemometec) was applied for 5 min. Then we added stabilization buffer (Solution 11, 910-3011, Chemometec) to the samples to stop the reaction, and cellular fluorescence was analyzed with the NucleoCounter NC-3000™ system (Chemometec). The ratio of the cells in the different cell cycle phases was determined based on the DNA content of the cells (Supplementary Figure S3).

### 2.5. Three-Dimensional Spheroid Assay

We seeded 5000 cells/well in 200 µL medium at the inner 60 wells of a 96-well plate coated with polyHEMA and filled the outer wells with PBS to reduce evaporation. In order to enhance single spheroid formation, we collected the cells at the bottom of the wells by centrifugation at 540× *g*. Next, we incubated the cells for 24 h, during which the cells aggregated into spheroids. Then we treated the spheroids by the addition of a further 100 µL medium supplemented with CUDC-907 (1 nM, 5 nM, 10 nM, 30 nM, 50 nM) as a single agent or in combination with Cisplatin (3 μM). Each treatment group contained five spheroids. After 72 h, we repeated the treatment. We removed 90 µL medium from each well and provided again 100 µL medium supplemented with the inhibitors. After an additional 72 h, each spheroid was photographed using an Echo Revolve R3 microscope camera on the sixth day of the treatment, and images were analyzed using Echo Revolve software (version 6.4.2). Briefly, the area of the 2D projections of the spheroids or the diameter were measured manually, and their values were used to calculate first the radius and then the volume of the spheroids using the formula V = 4/3 × π × radius^3^. Additionally, the viability of the cells in the spheroids was also determined at the end of the 6 day long treatment with Cell Counting Kit-8 assay (Sigma-Aldrich 96992). To each well 10 μL of CCK-8 solution was added, and the plate was incubated in the incubator for 24 h. Then absorbance was measured at 450 nm with the Infinite F50 Plate Reader, Tecan (Männedorf, Switzerland). All experiments were performed in three independent replicates.

### 2.6. Copy Number Variation Analysis

*MYC* copy number analysis was performed by using the FAM-labeled MYC TaqMan Copy Number Assay (product code Hs02758348_cn; Thermo Fisher Scientific, Waltham, MA, USA) and the VIC-labeled RNase P TaqMan Copy Number Reference Assay (product code 4403326; Thermo Fisher Scientific) as reference, as described earlier [39,40]. Briefly, genomic DNA was isolated from each PM cell line with the DNeasy Blood & Tissue Kit (Qiagen, Hilden, Germany) following the instructions in the manual. We measured DNA concentrations by using the Qubit^®^ 2.0 Fluorometer assay kits (LifeTechnologies, Carlbald, CA, USA). Then, 5 ng of gDNA was used as a template for qPCR using the Applied Biosystems StepOnePlus PCR equipment (Applied Biosystems, Foster City, CA, USA). All reactions were performed in duplicates, and nontemplate controls were included. Copy number was calculated by Copy Caller Software version 2.1 from Thermo Fisher Scientific.

## 3. Results

### 3.1. The Effect of CUDC-907 Treatment on PM Cells

**Figure 1:**
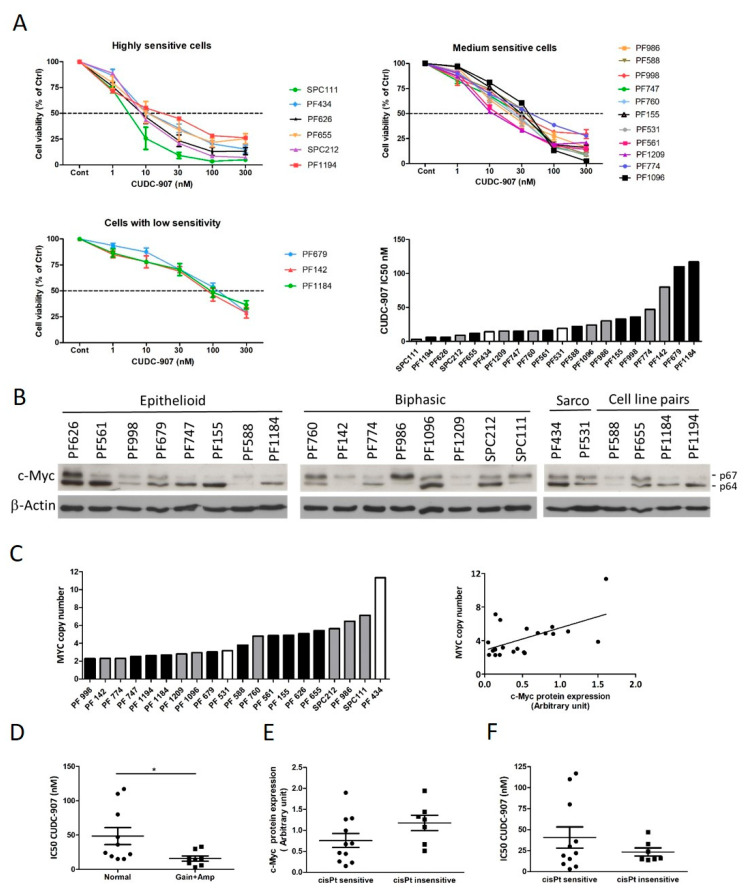
PM cell lines show strong sensitivity to CUDC-907 treatment. (**A**) Cell viability was measured with SRB assays after 72 h-long treatment, and half maximal inhibitory concentration (IC50) was calculated. Data points represent means ± SEM from three independent experiments. Colors represent the original tumor histotype: epithelioid: black, biphasic: gray, sarcomatoid: white. (**B**) Protein expression of c-Myc isoforms p67 and p64 was determined by Western blot analysis. Pictures show one representative experiment of two independent measurements; β-Actin was used as a loading control. (**C**) MYC gene copy number was analyzed in each cell line. Correlation with c-Myc protein expression was calculated with the Pearson test (r = 0.5185, *p* = 0.016). Colors represent the original tumor histotype: epithelioid: black, biphasic: gray, sarcomatoid: white. (**D**) The CUDC-907 IC50 values of the cell lines with MYC copy number gain or amplification were significantly lower than the cells with normal copy number. Statistical comparison was calculated by a two-tailed *t*-test with Mann–Whitney test (*p* = 0.0183, * *p* < 0.05). (**E**,**F**) c-Myc protein expression and CUDC-907 sensitivity in cisplatin sensitive and insensitive cell lines. This difference between the two groups did not reach statistical significance, (*p* = 0.1187 and *p* = 0.3089, respectively).

First, we analyzed the sensitivity of 20 PM cell lines to CUDC-907 treatment and found that in 17 cell lines, cell viability was strongly reduced (Figure 1A, Supplementary Table S2). In highly sensitive cells, this effect was evoked already by treatment concentration in the low nanomolar range, and in medium-sensitive cells by 15–30 nM concentrations. Only three cell lines showed substantially lower sensitivity with an IC50 value around 100 nM. We found no significant correlation between BAP1 or PTEN loss and CUDC-907 sensitivity. Since it was demonstrated in other cancer types that c-Myc expression was associated with stronger CUDC-907 sensitivity, we analyzed c-Myc protein expression level in our cell line panel (Figure 1B) and found that the expression and ratio of the c-Myc isoforms (p64, p67) showed a great variation among the cells. Elevated expression of Myc is often caused by increased copy number of the *MYC* gene. We analyzed the copy number of *MYC* in our cell line panel, and in seven cell lines we detected copy number gain (≥4), and in three cell lines amplification (≥6). *MYC* copy number and c-Myc protein expression showed significant correlation as expected (Figure 1C). Importantly, CUDC-907 sensitivity was significantly higher in cell lines with higher c-Myc expression due to *MYC* copy number gain or amplification (Figure 1D). We also examined whether there is a correlation between c-Myc expression, CUDC-907 sensitivity, and sensitivity to cisplatin treatment in the cell line cohort. We found that cell lines with higher c-Myc protein expression had a lower sensitivity to cisplatin, but the difference did not reach statistical significance (Figure 1E). Additionally, all cisplatin-insensitive cell lines were sensitive to CUDC-907, and the three cell lines with low CUDC-907 sensitivity show sensitivity to cisplatin (Figure 1F).

**Figure 2:**
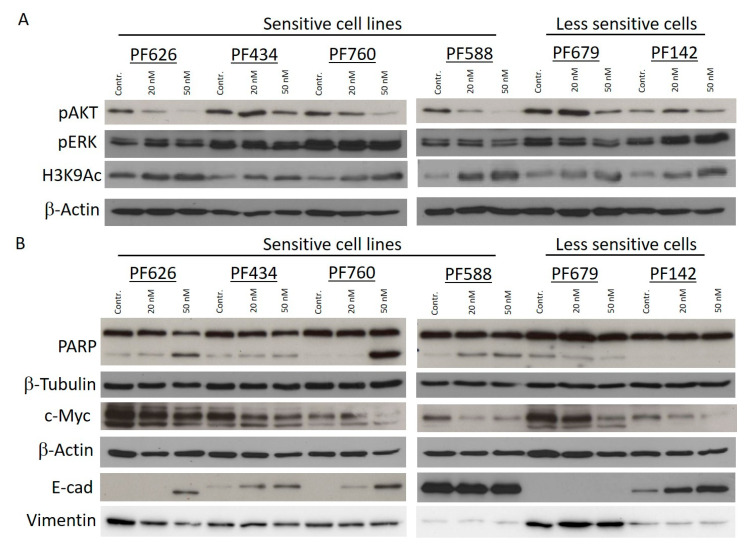
CUDC-907 treatment downregulates c-Myc and upregulates E-Cadherin expression in PM cells. (**A**) pAKT, pERK, and H3K9Ac expression levels were analyzed after 24-h long treatment. (**B**) PARP, c-Myc, E-Cadherin, and vimentin expression levels were analyzed after 72-h long treatment. Pictures show one representative experiment of two or three independent measurements; β-Actin and β-Tubulin were used as loading controls.

We further analyzed four sensitive and two less CUDC-907 sensitive PM cell lines after treatment with 20 and 50 nM CUDC-907. We found that histone acetylation on histone H3 at Lys9 residue was increased in all cell lines by both treatment concentrations, while AKT phosphorylation was strongly decreased in all sensitive cell lines, but it did not change in one of the less sensitive cells (Figure 2A). ERK activation was not influenced by the treatment. After 72 h long treatment formation of the apoptotic fragment of the PARP protein was present in three of the four sensitive cells but not in the less sensitive ones (Figure 2B). In certain cell lines, CUDC-907 treatment induced the expression of the cell adhesion molecule E-Cadherin, but we could observe the decrease in vimentin expression only in the PF626 cells. Importantly, c-Myc expression was reduced by the treatment in all cell lines. This might potentiate the effect of cisplatin as high c-Myc expression was also related to cisplatin sensitivity.

### 3.2. Combination Treatment with CUDC-907 and Cisplatin Shows Synergistic Effect

**Figure 3:**
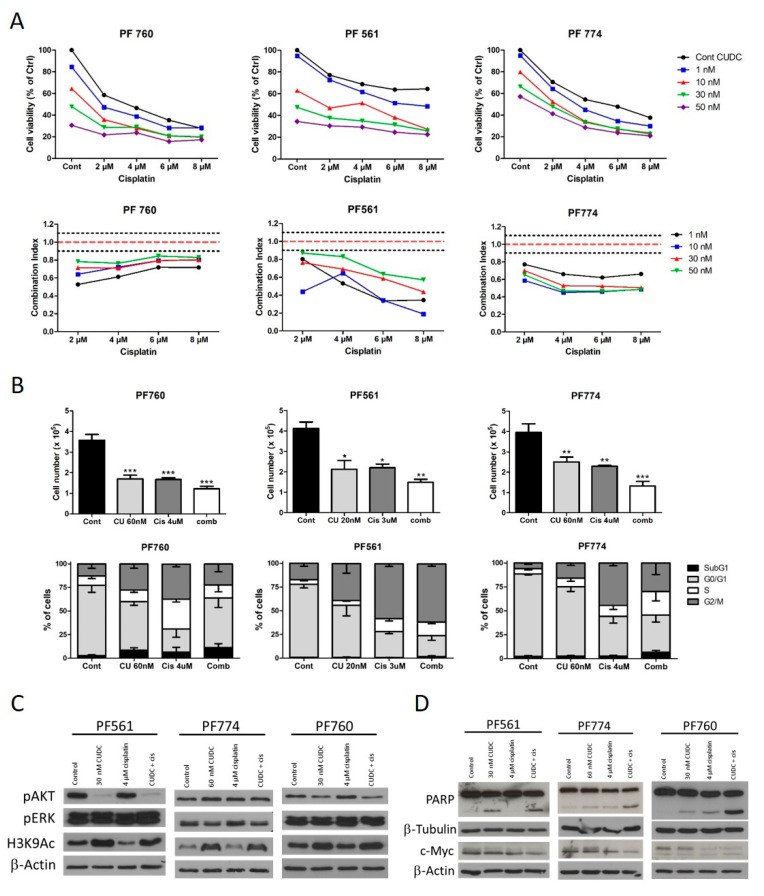
Combination treatment with CUDC-907 and cisplatin shows a synergistic effect. (**A**) The effect of combination treatments on cell viability was analyzed by the Sulforhodamine B assay. Cells were treated with increasing amounts of CUDC-907 and cisplatin for 72 h. Graphs show the average values of three independent experiments. The combination index (CI) was calculated for each combination, and it indicates synergism (CI < 0.9), additive effect (CI is between 0.9 and 1.1), or antagonism (CI > 1.1). Dotted lines show these cut-offs. (**B**) Cell number and percentage of cells in each cell cycle phase were determined after treatment with CUDC-907 and cisplatin for 72 h in the indicated concentrations. Bars represent means ± SEM from three independent experiments. Statistical significance was calculated with repeated measures ANOVA with a Bonferroni post hoc test (* *p* < 0.05, ** *p* < 0.01, *** *p* < 0.001). (**C**,**D**) pAKT, pERK, and H3K9Ac expression levels were analyzed after 24 h long treatment. PARP and c-Myc expression levels were analyzed after 72 h long treatment. Pictures show one representative experiment of two or three independent measurements; β-Actin and β-Tubulin were used as loading controls.

In order to assess the effect of the combination treatment with CUDC-907 and cisplatin on PM cells, we have chosen three cell lines that have moderate sensitivity to both drugs. First, we performed a viability assay and found that in all three cell lines, the combination treatment had a synergistic effect (Figure 3A). Then, with a selected concentration pair to analyze the changes in cell number and cell cycle distribution, we found that cell number was further reduced by the combination treatment compared to the single treatments in all three cell lines (Figure 3B). In PF774 and PF760 cells, combination treatment induced cell death (SubG1) besides G2/M and S phase arrest, while in PF561 cells, both cisplatin and the combination treatment evoked G2/M phase arrest in the majority of the cells. Immunoblot analysis revealed that in both PF561 and PF760 cells, AKT phosphorylation was reduced and H3 histone acetylation was increased by CUDC-907, both in single and combination treatments. Interestingly, in the PF774 cells, which have lower sensitivity to CUDC-907, AKT activation was not modified by any of the treatments, but increased histone acetylation was present. The cleaved fragment of PARP was increased most pronounced by the combination treatment in PF760 and PF774 cells, while in PF561 cells it was detected in a similar amount in the cisplatin and the combination-treated samples. c-Myc expression was reduced by cisplatin and combination treatment in PF774 and PF760 cells, and it did not change by any of the treatments in PF561 cells.

### 3.3. Cisplatin-Resistant Cell Line Has Increased CUDC-907 Sensitivity

**Figure 4:**
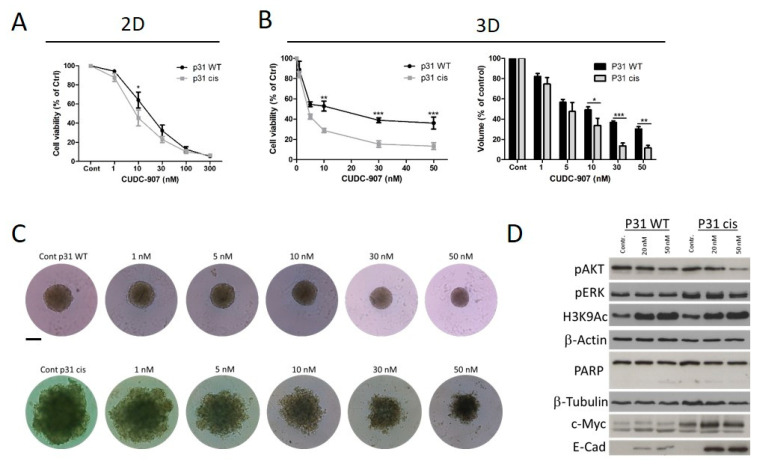
Cisplatin-resistant cell line has increased CUDC-907 sensitivity. (**A**) The CUDC-907 sensitivity of the cisplatin-sensitive (P31 WT) and cisplatin-resistant (P31 cis) cell line pair was analyzed by Sulforhodamine B assay. Cells were treated with increasing amounts of CUDC-907 for 72 h. The graph shows the average values of three independent experiments. Statistical significance was calculated with repeated measures ANOVA with Bonferroni post hoc test (*p* < 0.05). (**B**) Tumor cell spheroids were treated with CUDC-907 for 6 days. Cell viability was analyzed by CCK-8 assay, and spheroid volume was calculated from the area of 2D projections. Bars represent means ± SEM from three independent experiments. Statistical significance was calculated with repeated measures ANOVA with Bonferroni post hoc test (* *p* < 0.05, ** *p* < 0.01, *** *p* < 0.001). (**C**) Representative phase contrast images (10× objective) of tumor cell spheroid after CUDC-907 treatment for 6 days. The scale bar is 200 μm. (**D**) pAKT, pERK and H3K9Ac expression levels were analyzed after 24 h long treatment. PARP, c-Myc and E-Cadherin expression levels were analyzed after 72 h long treatment. Pictures show one representative experiment of two or three independent measurements; β-Actin and β-Tubulin were used as loading controls.

To further analyze the interplay between cisplatin and CUDC-907 sensitivity of PM cells, we used a cell line pair that contained the parental cell line P31 WT with an IC50 of 4.4 μM for cisplatin and the resistant cell line P31 cis with an IC50 of 38 μM. We found that in the 2D cell viability assay, the cisplatin-resistant cells showed a slightly higher sensitivity to CUDC-907 treatment (P31 WT IC50 is 16 nM, while P31 cis IC50 is 12 nM; Figure 4A). The difference reached significance at the 10 nM treatment. Using the same categorization as in Figure 1, P31 cis belongs to the highly sensitive group of cells and P31 WT to the medium sensitive ones. This difference was more pronounced when we compared the two cell lines’ sensitivity after the formation of 3D spheroids. Cell viability was significantly reduced in the cisplatin-resistant cells upon 10, 30, and 50 nM treatments, and spheroid volume also showed a more pronounced decrease (Figure 4B and Supplementary Figure S4). Importantly, the morphology of the spheroids was different in the two cell lines. P31 WT formed more compact, smaller spheroids with a smooth surface, while the spheroids from p31 cis cells were larger with an irregular surface (Figure 4C). Protein expression analysis showed that c-Myc expression was highly abundant in both cell lines, but it was even higher in the cisplatin-resistant cells. CUDC-97 treatment did not induce significant changes in the expression of c-Myc (Supplementary Figure S5). In contrast, the protein level of E-cadherin strongly increased in P31 cis cells, and it was only slightly elevated in the P31 WT cell line. CUDC-907 treatment moderately decreased AKT activation and increased histone H3 acetylation, but it did not alter the level of pERK (Supplementary Figure S5).

**Figure 5:**
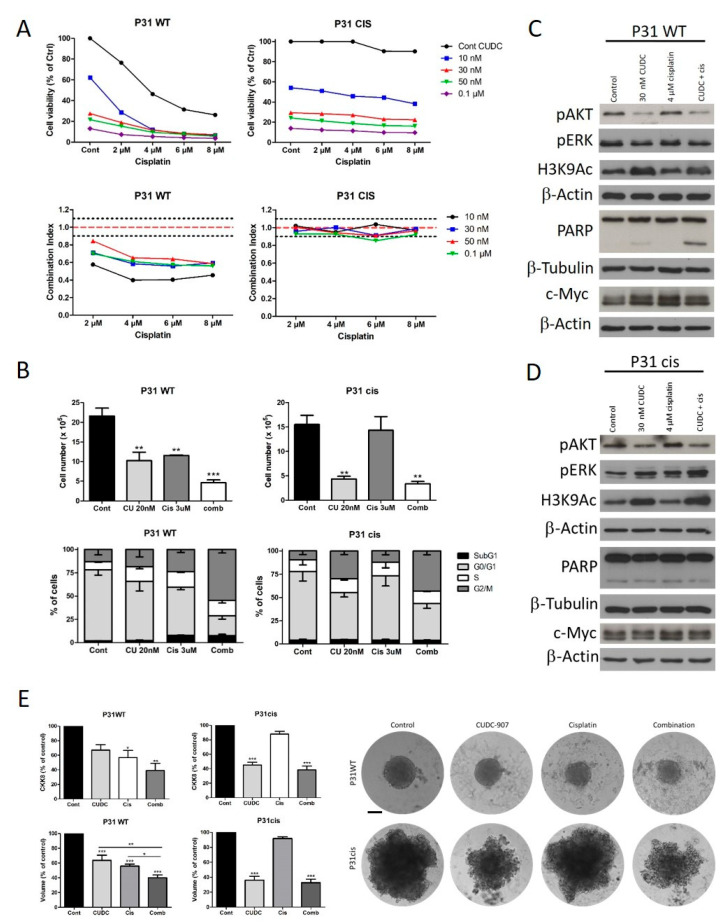
Combination treatment with cisplatin synergistically reduces cell growth through G2/M arrest in both cisplatin-sensitive and resistant cells. (**A**) Cells were treated with increasing amounts of CUDC-907 and cisplatin for 72 h, and cell viability was analyzed by SRB assay. Graphs show the average values of three independent experiments. The combination index (CI) was calculated for each combination, and it indicates synergism (CI < 0.9), additive effect (CI is between 0.9 and 1.1), or antagonism (CI > 1.1). Dotted lines show these cut-offs. (**B**) Cell number and percentage of cells in each cell cycle phase were determined after treatment with CUDC-907 and cisplatin for 72 h in the indicated concentrations. Bars represent means ± SEM from three independent experiments. Statistical significance was calculated with repeated measures ANOVA with a Bonferroni post hoc test. (**C**,**D**) pAKT, pERK, and H3K9Ac expression levels were analyzed after 24 h long treatment. PARP and c-Myc expression levels were analyzed after 72 h long treatment. Pictures show one representative experiment of two or three independent measurements; β-actin and β-tubulin were used as loading controls. (**E**) Tumor cell spheroids were treated with CUDC-907 (10 nM) or cisplatin (3 μM) alone or in combination for 6 days. Spheroid volume was calculated from the diameter of 2D projections. Bars represent means ± SEM from three or four independent experiments. Statistical significance was calculated with repeated measures ANOVA with Bonferroni post hoc test (* *p* < 0.05, ** *p* < 0.01, *** *p* < 0.001). Representative phase contrast images (10× objective) of tumor cell spheroid after 6 days of treatment. Scale bar is 160 μm.

We also analyzed the effect of CUDC-907 in combination with cisplatin on the P31 cell line pair. In the case of the parental cell line, the combination treatment had a strong synergistic effect on cell viability, while in the cisplatin-resistant cells, it had an additive effect (Figure 5A). In the cisplatin-sensitive cells, both CUDC-907 and cisplatin decreased cell number and increased the ratio of the cells in the S and the G2/M phase, and this was strongly enhanced by the combination of the two drugs. In the P31 cis cells, a similar effect was initiated by CUDC-907 alone and the combination treatment, while cisplatin alone did not cause any alterations (Figure 5B). The ratio of the cells in the sub-G1 cell fraction was increased by the single cisplatin and the combination treatment in the P31 WT cells, but it remained similar in all samples of the P31 cis cells. Protein analysis showed that AKT activation decreased and histone H3 acetylation increased in both cell lines after CUDC-907 and combination treatment, but an increase in the apoptotic fragment of PARP was detected only in the parental cell line after combination treatment. c-Myc expression was not altered by the treatments in either cell line (Figure 5C,D). We also analyzed the influence of this combination treatment on the spheroid growth of P31 WT and P31 cis cells. We found that in the P31 WT cell, it synergistically reduced both spheroid volume and cell viability. In the case of the cisplatin-resistant P31 cells, CUDC-907 treatment had a similar effect as CUDC-907 treatment alone (Figure 5E, Supplementary Figure S6). Our results show that the combination treatment of CUDC-907 and cisplatin could be an effective therapy for cisplatin-sensitive tumors.

## 4. Discussion

In our work, we investigated, for the first time, the effect of the dual inhibitor CUDC-907 on PM cells. We analyzed the sensitivity of 22 PM cell lines to CUDC-907 treatment and found that the half maximal inhibitory concentration (IC50) for most cell lines was between 3 and 50 nM. This concentration could be clinically relevant as the plasma concentration of CUDC-907 was around 22 nM in a phase 1 study of DLBCL patients [25]. We found no correlation between CUDC-907 sensitivity and the histopathological subtype of the original tumor or age, or BAP1 mutational status in our cell line cohort. Previous studies showed that increased c-Myc expression is frequent in PM tumors due to increased gene copy number [16]. Our analysis supported these findings as in seven cell lines we detected copy number gain (≥4) and in three cell lines amplification (≥6). We found that—similarly to several other cancer types—higher c-Myc expression was a predictive factor for CUDC-907 sensitivity and the treatment often downregulated c-Myc protein expression [13]. Interestingly, the ratio of the two c-Myc isoforms p64 and p67 varied strongly among the cell lines. The two proteins differ in their initiation of translation, and they have distinct functional roles. A growth inhibitory role is attributed to p67, while p64 has a growth stimulatory effect [41]. We found that CUDC-907 treatment affected the expression of the two isoforms differently in certain cell lines, an observation that needs to be further explored. Combination treatment with cisplatin had a synergistic effect in the investigated cell lines, except in the cisplatin-resistant cell line model P31 cis; however, increased growth inhibition and cell cycle arrest were initiated even in this cisplatin-resistant model. This is similar to the findings in SCLC cell lines, where elevated c-Myc expression was coupled with platinum resistance, and CUDC-907 decreased Myc expression and sensitized the cells to platinum and etoposide treatment [17]. In the control and cisplatin-resistant cell line pair investigated in our study, c-Myc expression was higher in the cisplatin-resistant cells, and CUDC-907 treatment synergistically induced G2/M arrest in these cells. It was described earlier that CUDC-907 could restore cisplatin sensitivity in cancer cells by decreasing ABCC2 expression, resulting in the accumulation of platinum drugs in the cells and increased cell cycle arrest [42]. Recently, it was found that mesothelioma cells with high c-Myc expression show intrinsic resistance to novel TEAD inhibitors [43]. Downregulation of c-Myc expression by CUDC-907 might potentiate the effect of these inhibitors as well. We found that CUDC-907 decreased PM cell growth also in 3D spheroids. In HCC cell lines, CUDC-907 was identified as a potent inhibitor with a spheroid-based drug screening test. It was reported that it decreased cell viability of HCC cell lines and primary HCC cells with an IC50 between 4 and 20 nM, similarly to our results, while primary hepatocytes were not affected by the treatment [19]. In another study, a bladder cancer cell line and its cisplatin-resistant pair were used, and CUDC-907 treatment reduced cell viability and induced cell death in both cell lines. Interestingly, 3D spheroid cells were less sensitive to CUDC-907 treatment than in 2D cultures, but we have not observed this in our PM model [20]. They also described that CUDC-907 treatment strongly increased the mRNA expression of E-cadherin and reduced the expression of vimentin and MMP-2, suppressing epithelial–mesenchymal transition in this way. Upregulation of E-Cadherin and downregulation of β-catenin, Slug, and Snail after CUDC-907 treatment were also found in esophageal squamous cell carcinoma cells [18]. In certain cell lines, we also detected the upregulation of E-cadherin upon CUDC-907 treatment; interestingly, this was not dependent on the sensitivity of the cell line to the inhibitor. In one out of the six studied cell lines, vimentin expression was also decreased by CUDC-907 treatment. In DLBCL cell lines CUDC-907 decreased the activation of PI3K targets like AKT, S6 and 4EBP1 and elevated the acetylation level of histone 3 [44]. Similarly, we found in PM cell lines that CUDC-907 treatment reduced pAKT level and increased histone 3 acetylation. In lung cancer cells, CUDC-907 initiated G2/M phase arrest through reducing the expression of cell cycle regulatory proteins as Cdc25C, CdC2, and Cyclin B1, and by elevating p21 protein level. Also, the treatment induced abnormal mitosis in these cells through the decrease in Aurora A and B and polo-like kinase protein levels [45]. They found that CUDC-907 exerted these effects through the activation of the JNK/p38 mitogen-activated protein kinase pathway and through the inhibition of the YAP/TAZ signaling. In PM cell lines, CUDC-907 treatment also induced cell cycle arrest, and it strongly reduced cell proliferation. In thyroid cancer cell lines, where both the PI3K/AKT and RAF/RAS/MEK/ERK pathways are strongly activated due to frequent oncogene mutations, CUDC-907 treatment decreased both pAKT and pERK levels, resulting in growth inhibition in both in vitro and in vivo models [46]. Interestingly, we found that in PM cell lines, AKT activation was decreased by the treatment, but pERK level was not altered in any of the cell lines.

## 5. Conclusions

Our results show that most PM cell lines are sensitive to CUDC-907, and the combination with cisplatin treatment can lead to synergistic effects. For this reason, CUDC-907 might be a potent agent in pleural mesothelioma treatment. Our findings provide a rationale for the clinical exploration of CUDC-907 either in the first-line setting in combination with platinum-based chemotherapy in selected cases with MYC amplification or after progression on first-line standard-of-care chemotherapy.

## Data Availability

The raw data supporting the conclusions of this article will be made available by the authors on request.

## Supplementary Materials

The following supporting information can be downloaded at: https://www.mdpi.com/article/10.3390/cells14201599/s1, Figure S1: Representative phase contrast images of the PM cell lines; Figure S2: Representative phase contrast images of the PM cell lines; Figure S3: Representative gating strategy to identify the cell cycle phases; Figure S4: Tumor cell spheroids were treated with CUDC-907 for 6 days. Spheroid volume was calculated from the area of 2D projections; Figure S5: Densitometry analysis of c-Myc and H3K9Ac protein expression level after treatment with CUDC-907 for 72 or 24 hours; Figure S6: Tumor cell spheroids were treated with CUDC-907 (20 nM) and cisplatin (3 mM) alone or in combination for 6 days; Table S1: Patient characteristics and BAP1/PTEN status of the cell line cohort; Table S2: CUDC-907 and cisplatin sensitivity of PM cell lines.
