# Supplementary material for: Dual HDAC and PI3K Inhibitor CUDC-907 Inhibits Growth of Pleural Mesothelioma: The Impact of Cisplatin Sensitivity and Myc Expression
Source: Cells. 2025 Oct 15;14(20):1599. doi: 10.3390/cells14201599 (PMC12563042; doi:10.3390/cells14201599)
Supplement: Supplementary file 1 [file cells-14-01599-s001.zip › cells-3849412-supplementary.pdf]

Supplementary Tables

Cell line	Age (years)	Gender	Asbestos exposure	Disease course	Histology	BAP1 expression	PTEN expression
PF 142	64	female	yes	diagnostic	biphasic	no	yes
PF 155	71	male	n. a.	diagnostic	epithelioid	no	no
PF 434	86	male	yes	diagnostic	sarcomatoid	yes	yes
PF 531	57	male	yes	diagnostic	sarcomatoid	yes	yes
PF 561	78	male	yes	diagnostic	epithelioid	yes	yes
**PF 588**	64	female	yes	diagnostic	epithelioid	no	yes
PF 626	74	male	yes	diagnostic	epithelioid	yes	yes
**PF 655**	65	female	yes	post-chemo	epithelioid	no	yes
PF 679	64	male	yes	post-therapy	epithelioid	yes	no
PF 747	77	male	yes	post-therapy	epithelioid	yes	yes
PF 760	83	male	yes	diagnostic	biphasic	no	yes
PF 774	44	female	no	diagnostic	biphasic	yes	yes
PF 986	64	male	no	diagnostic	biphasic	yes	yes
PF 998	71	male	yes	post-therapy	epithelioid	yes	yes
PF 1096	62	male	yes	diagnostic	biphasic	yes	yes
**PF 1184**	73	male	yes	diagnostic	epithelioid	no	yes
**PF 1194**	73	male	yes	post-chemo	epithelioid	no	yes
PF 1209	57	male	yes	diagnostic	biphasic	yes	no

Supplementary Table S1. Patient characteristics and BAP1/PTEN status of the cell line cohort. PF588 - PF655 and PF1184 – PF1194 are cell line pairs derived from the same patients. BAP1 and PTEN loss were determined by western blot analysis. (n.a. – not available)

**Cell lines**	**CUDC-907 IC50 (nM)**	**Cisplatin IC50 (μM)**
SPC111	3	4,17
PF1194	6	5,8
PF626	6	4,4
SPC212	9	1,7
**PF655**	12	0,75
PF434	14	5,4
PF1209	15	2,4
PF747	15	>8
PF760	15	5,9
PF561	16	>8
p31 WT	16	4,4
PF531	19	0,56
**PF588**	22	3,93
PF1096	24	7,8
PF986	30	2,7
PF155	33	5,5
PF998	36	1,2
PF774	47	6,7
PF142	80	0,52
PF679	110	2,3
PF1184	117	4,75

Supplementary Table S2. CUDC-907 and cisplatin sensitivity of PM cell lines. IC50 values were calculated based on the SRB cell toxicity test.

Supplementary Figures

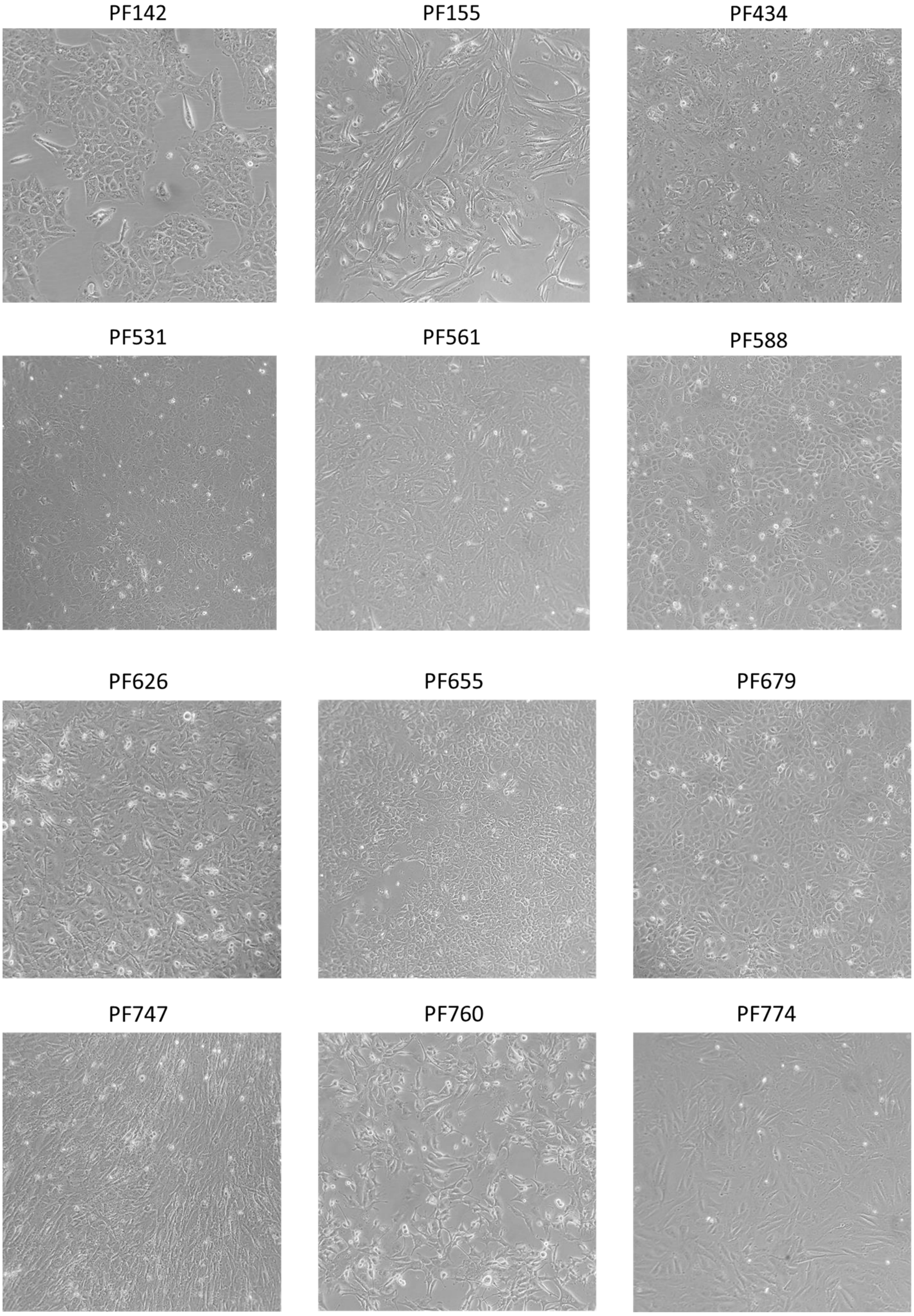

Supplementary Figure S1. Representative phase contrast images of the PM cell lines.

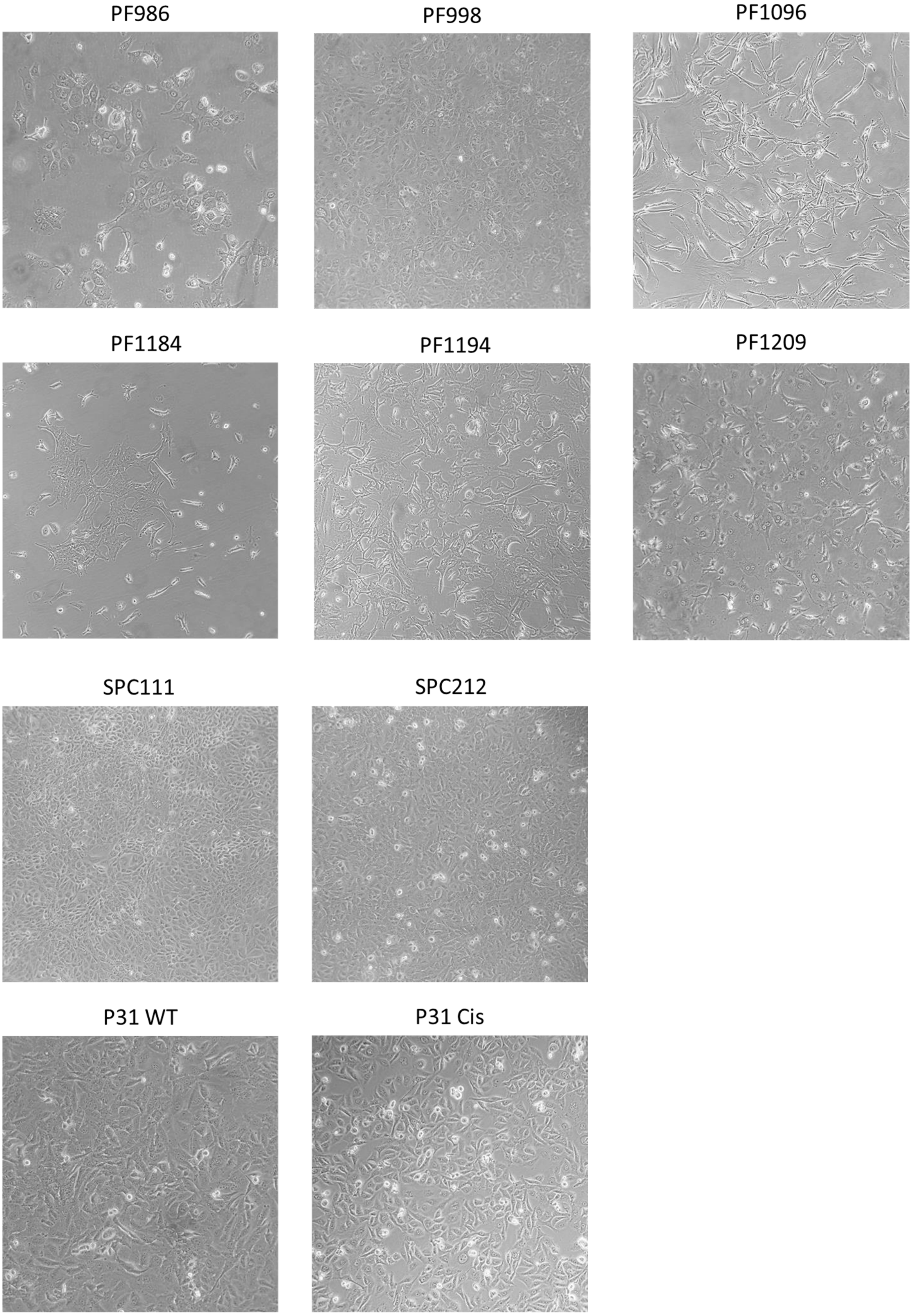

Supplementary Figure S2. Representative phase contrast images of the PM cell lines.

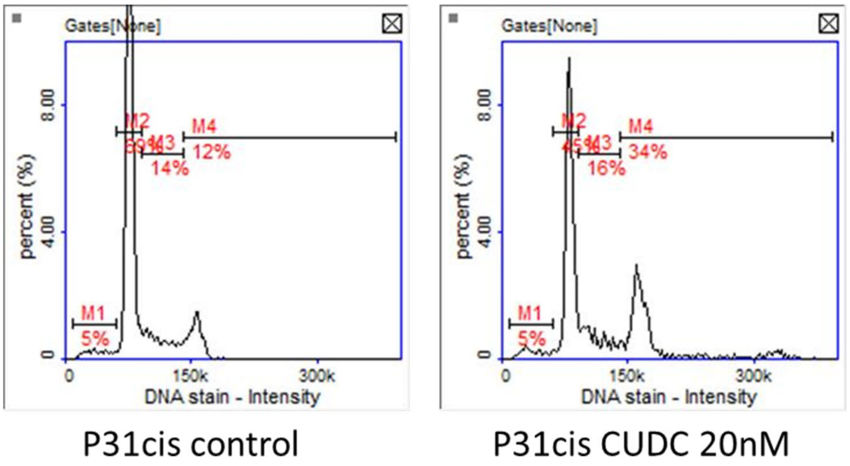

Supplementary Figure S3. Representative gating strategy to identify the cell cycle phases. M1: sub-G1 phase, M2: G0/G1 phase, M3: S phase, M4: G2/M phase.

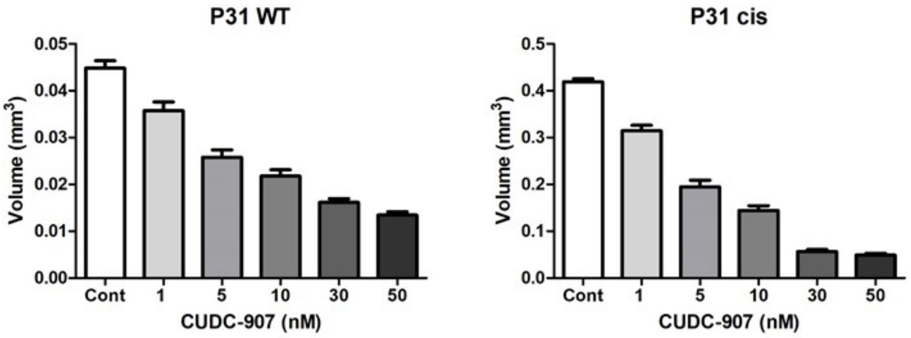

Supplementary Figure S4. Tumor cell spheroids were treated with CUDC-907 for 6 days. Spheroid volume was calculated from the area of 2D projections. Bars represent means ± SEM from three independent experiments.

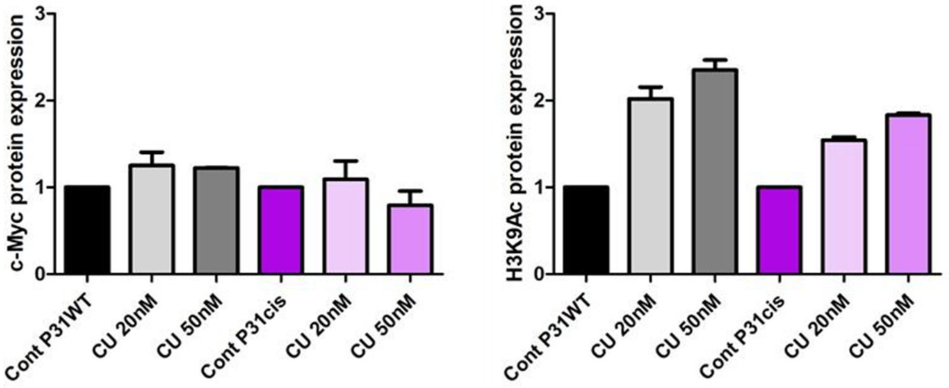

Supplementary Figure S5. Densitometry analysis of c-Myc and H3K9Ac protein expression level after treatment with CUDC-907 for 72 or 24 hours, respectively.

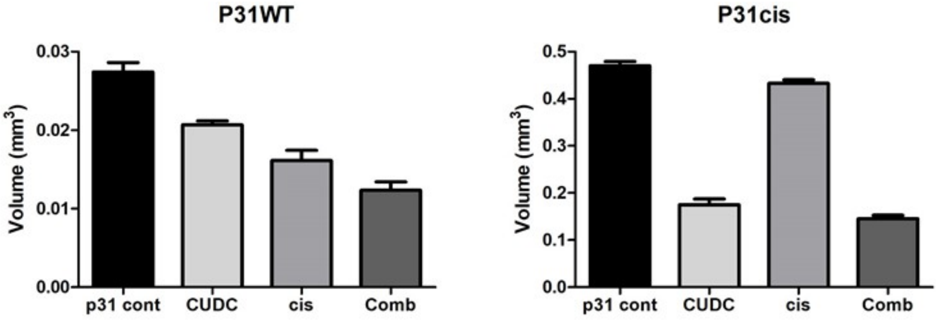

Supplementary Figure S6. Tumor cell spheroids were treated with CUDC-907 (20 nM) and cisplatin (3 μM) alone or in combination for 6 days. Spheroid volume was calculated from the area of 2D projections. Bars represent means ± SEM from three independent experiments.
